# A Deep-Learning-Based Algorithm for Landslide Detection over Wide Areas Using InSAR Images Considering Topographic Features

**DOI:** 10.3390/s24144583

**Published:** 2024-07-15

**Authors:** Ning Li, Guangcai Feng, Yinggang Zhao, Zhiqiang Xiong, Lijia He, Xiuhua Wang, Wenxin Wang, Qi An

**Affiliations:** School of Geosciences and Info-Physics, Central South University, Changsha 410083, China; ning_li@csu.edu.cn (N.L.); yinggang.zhao@hirain.com (Y.Z.); zqxiong_flhs@csu.edu.cn (Z.X.); lijiahe@csu.edu.cn (L.H.); csuxhgd@csu.edu.cn (X.W.); wangwx@csu.edu.cn (W.W.); anqi27@csu.edu.cn (Q.A.)

**Keywords:** landslide detection, topographic features, InSAR, semantic segmentation

## Abstract

The joint action of human activities and environmental changes contributes to the frequent occurrence of landslide, causing major hazards. Using Interferometric Synthetic Aperture Radar (InSAR) technique enables the detailed detection of surface deformation, facilitating early landslide detection. The growing availability of SAR data and the development of artificial intelligence have spurred the integration of deep learning methods with InSAR for intelligent geological identification. However, existing studies using deep learning methods to detect landslides in InSAR deformation often rely on single InSAR data, which leads to the presence of other types of geological hazards in the identification results and limits the accuracy of landslide identification. Landslides are affected by many factors, especially topographic features. To enhance the accuracy of landslide identification, this study improves the existing geological hazard detection model and proposes a multi-source data fusion network termed MSFD-Net. MSFD-Net employs a pseudo-Siamese network without weight sharing, enabling the extraction of texture features from the wrapped deformation data and topographic features from topographic data, which are then fused in higher-level feature layers. We conducted comparative experiments on different networks and ablation experiments, and the results show that the proposed method achieved the best performance. We applied our method to the middle and upper reaches of the Yellow River in eastern Qinghai Province, China, and obtained deformation rates using Sentinel-1 SAR data from 2018 to 2020 in the region, ultimately identifying 254 landslides. Quantitative evaluations reveal that most detected landslides in the study area occurred at an elevation of 2500–3700 m with slope angles of 10–30°. The proposed landslide detection algorithm holds significant promise for quickly and accurately detecting wide-area landslides, facilitating timely preventive and control measures.

## 1. Introduction

Landslide is a geological hazard that occurs on mountain slopes caused by gravity, earthquake, river erosion, rainfall, and human activities [1,2]. Due to the joint effects of human activities and climate change, the occurrence of landslides has become increasingly frequent, threatening the safety of people’s lives and engineering projects [3,4,5]. Therefore, the effective identification and monitoring of landslides are important. Traditional landslide detection methods rely on labor-intensive field investigation. Despite its high accuracy, it is impractical for large-scale identification due to high costs and low efficiency. The precise leveling and GPS were developed to provide landslide-related deformation information and deformation characteristics, but they are limited to monitoring single or small-scale landslides that have been identified [6,7,8]. Nowadays, remote sensing data have been widely applied in landslide prediction, monitoring, and identification [9]. And due to its wide data coverage, high precision, and short revisit cycle, remote sensing has great potential to overcome the drawbacks of being time-consuming, laborious, and costly in identifying and monitoring wide-area landslides [10]. The Interferometric Synthetic Aperture Radar (InSAR) technique can obtain detailed surface deformation from SAR images, playing an important role in wide-area landslide identification and research [11,12,13].

With the advancement of machine learning, it has been combined with remote sensing data to improve the accuracy and reliability of landslide identification [14]. Deep learning, a branch of machine learning, uses multi-layer nonlinear information processing to learn information from datasets for feature extraction, transformation, and classification [15]. The proliferation of SAR satellites has resulted in vast amounts of accumulated data for research purposes. Consequently, InSAR big data can provide sufficient training samples for the deep learning model, enhancing the model’s generalizability and enabling them to better understand, learn, and extract deformation information from InSAR data [16]. In landslide identification, deep learning methods offer higher efficiency and faster speed and are less susceptible to subjective influences compared to traditional visual interpretation methods. Therefore, many studies have applied deep learning methods to optical remote sensing images for landslide identification research [14,17,18,19]. Optical images exhibit distinct spectral and textural changes before and after landslides, facilitating their differentiation from the surrounding terrain. However, landslides triggered by different geological environments may exhibit varying characteristics in optical remote sensing images [20]. Moreover, optical images can only identify landslides that have already occurred or have significant deformation but cannot identify creeping or unstable landslides effectively [21]. In contrast, InSAR can monitor small-scale deformations within a large area, which is an effective method to identify and discover landslides that are undergoing slow creep deformation. Therefore, the use of InSAR to obtain fine surface deformation and deep learning to identify ongoing or occurred landslides over wide areas has become a focus of current landslide identification research [22].

Currently, some studies have been conducted on InSAR geological hazard detection based on deep learning. The studies on landslide identification using InSAR data mainly fall into two categories: object detection and semantic segmentation [23,24]. Object detection aims to identify objects within images and determine their location and category. Fu et al. [25] combined phase-gradient stacking with a deep learning network based on YOLOv3 to automatically detect slow-moving landslides from large-scale interferograms, improving the efficiency of landslide identification. Semantic segmentation, on the other hand, classifies each pixel in the image, enabling precise segmentation of the image to determine the boundaries of different regions or objects. Chen et al. [26] designed a semantic segmentation network called deep residual shrinkage U-Net (DRs-UNet), which is proved to be able to effectively identify active landslides automatically from InSAR images. However, the application of deep learning methods is still constrained by factors such as model applicability, the volume of training samples, and differences in InSAR data types. To improve landslide identification accuracy, scholars are continuously exploring new methods.

The InSAR results include many types of deformation, such as landslide deformation, ground subsidence, mining-induced deformation, and groundwater-related deformation [27]. Solely using InSAR deformation results for landslide identification over wide areas may inadvertently include other types of ground movement, thus affecting the accuracy of landslide identification. In addition, the occurrence of landslides is influenced not only by natural factors such as geological environment and rainfall but is also closely related to terrain features [28,29]. In this study, we address this issue by integrating topographic data through feature fusion. We improve the DeforNet network, a geological hazard identification model, and propose a multi-source data fusion network based on DeforNet (MSFD-Net). This is a semantic segmentation network. Compared to object detection networks, semantic segmentation can not only locate the specific position of landslides but also delineate their extent and calculate their area. This facilitates the compilation of landslide inventories over wide areas. To validate the efficacy of our approach, we apply the network to detect the landslides in the middle and upper reaches of the Yellow River in eastern Qinghai Province. We also analyze the landslide distribution in the study area. Finally, we discuss the identification results and influencing factors of the network.

## 2. Methods

### 2.1. A Multi-Source Data Fusion Network Based on DeforNet

DeforNet proposed by Zhao et al. [30] in 2022 is a semantic segmentation network, tailored for detecting small- and medium-sized geological hazards from InSAR data. The network has a U-shaped encoder–decoder structure and adds depth-wise separable convolution modules [31] and convolutional block attention modules (CBAMs) [32]. It can assign different weights to deformation and non-deformation areas in the InSAR-wrapped map, enabling the extraction of accurate deformation features. However, we cannot directly apply Zhao et al.’s strategy for landslide identification, due to the similarity in deformation characteristics among different types of geological hazards in InSAR data.

**Figure 1:**
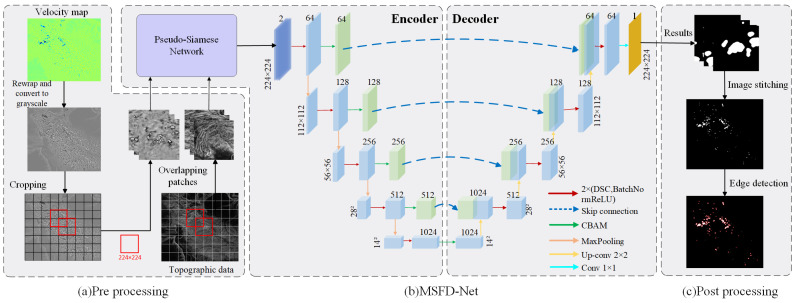
A landslide intelligent detection framework integrating topographic data. (**a**) The pretreatment of deformation results, (**b**) a multi-source data fusion network based on DeforNet (MSFD-Net), and (**c**) the post-processing of identification results.

When interpreting InSAR deformation results, experts often combine topographic features to determine the geological disaster types. For example, slope, aspect, and elevation are commonly used for landslide identification. Therefore, we chose to integrate InSAR deformation data with topographic data for landslide detection. And we improved the DeforNet network for geological hazard identification, constructing a new landslide detection strategy to enhance landslide identification accuracy (as shown in Figure 1). This strategy consists of three parts: (a) Data preprocessing, including rewrapping InSAR deformation rate results, converting them into grayscale images, and data segmentation. (b) A multi-source data fusion network based on DeforNet (MSFD-Net), which is primarily composed of a pseudo-Siamese network and the DeforNet semantic segmentation network. (c) Data post-processing, including result stitching and edge detection.

**Figure 2:**
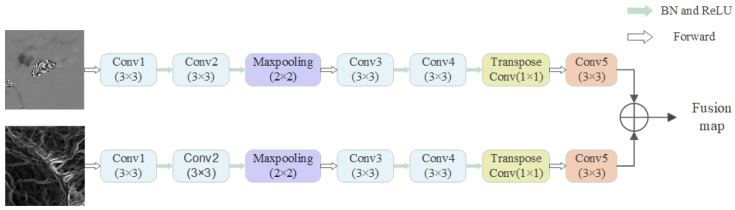
Pseudo-Siamese network structure.

The methods of multi-source data fusion are diverse and plentiful. Instead of using the traditional method of directly concatenating the deformation map and topographic data into dual-channel data, we first employed two independent single-channel feature extraction modules to separately extract texture features from landslide samples and topographic features from topographic data. Then, we fused the feature maps of the two modules at a higher level through feature map concatenation (Formula (1)). This feature extraction and fusion network is a pseudo-Siamese network without shared weights, which has shown good performance in detecting landslides by fusing optical images and DEM data [33]. As shown in Figure 2, the network comprises two identical convolutional neural networks (CNNs) that can be used for feature extraction. The purpose of using this network is to enhance the model’s ability to detect landslides. The CNN is designed to be lightweight and can serve as a common feature extractor, while also being well-integrated with other networks. In this network, we employed batch normalization (BN) and Rectified Linear Unit (ReLU) activation, which stabilize the network gradient propagation and accelerate the training speed. We used feature map concatenation for feature fusion, and the formula is as follows:

(1) $$T_{fusion}=Concat(\varphi_{1}\left(I_{wrap}\right),\;\varphi_{2}\left(I_{slope}\right))$$

where $\varphi_{1}$ and $\varphi_{2}$ represent two CNN feature extractors. $I_{wrap}$ and $I_{slope}$ are the input data. $T_{fusion}$ represents the fused feature map.

DeforNet includes convolution blocks and CBAMs in each layer of the encoder section. We use the fused feature map ($T_{fusion}$) obtained through the pseudo-Siamese network as the input for the DeforNet. First, we process it through two depth-wise separable convolutions and batch normalizations to extract the features of the feature map (dark red arrow). Then, the CBAM module assigns different weights to signals in the feature map (green arrow). The CBMA combines spatial and channel attention modules, where the channel attention module identifies the significant portions of the input feature map, highlighting meaningful features within each channel, while the spatial attention module determines regions of information richness across the feature map. These two modules are complementary, achieving better results than using a single module alone. Then, through the skip connection (blue dotted line), the weighted feature map is passed to the decoder and spliced with the feature map of the corresponding layer. The purple arrow represents the down-sampled pooling layer, which is used for feature dimension reduction to reduce over-fitting. Each layer of the decoder consists of bilinear up-sampling, skip connection, and convolution blocks. The bilinear up-sampling (orange arrow) ensures that the output of the feature map has the same size as the corresponding input layer. Skip connection fuses the weight with the corresponding up-sampling abstract features, retaining more detailed information and improving the accuracy of semantic segmentation. The convolution block in the decoder mirrors that in the encoder. The final classification layer of the network is a 1 × 1 convolution layer (light blue arrow), which outputs the prediction results, delineating the location of the landslide in the wrapped deformation map.

### 2.2. Network Training

**Figure 3:**
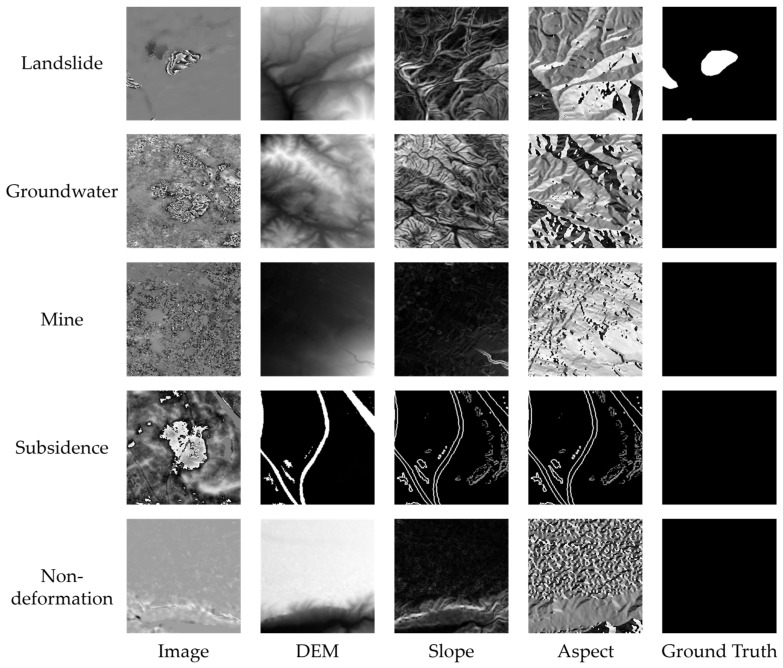
Positive and negative samples in the dataset.

To assess and validate the performance of our network, we constructed a sample dataset for training. This dataset comprises positive samples of landslide deformation and negative samples of ground subsidence deformation, mine-induced deformation, groundwater, and non-deformation areas (Figure 3). All these samples were collected based on previous experiments [12,30,34,35,36]. Previous studies have conducted experiments using CNN to assess the detection capabilities of volcanic deformation across different wrap intervals [37]. They propose a method that combines different wrap intervals to enhance the deformation detection capabilities of CNNs. Through experiments, Zhao et al. [30] further validated that using different wrap intervals for different types of geological hazards can effectively enhance the signal-to-noise ratio of the samples, thereby reducing the impact of noise. Therefore, we obtained the average deformation rates of the samples and then generated the wrapped deformation results of the samples through rewrapping. To ensure the ground truth of the positive landslide samples, we drew geohazard boundaries and generated binary labels based on the obtained landslide vector results, combined with expert interpretation and manual mapping.

We resampled the SRTM DEM data to the same resolution as the image and obtained slope maps and slope aspect maps through calculation. Since the network used in this study is based on single-channel data, we converted the topographic data into grayscale images. We took the midpoint of each landslide as the sample center, cropped the data into smaller sections of size 224 × 224 pixels, and created the landslide sample dataset. Each positive sample in the dataset contains at least one landslide. Due to the limited training samples, to avoid over-fitting and improve the generalization and robustness of the model, this study used sample augmentation methods, such as rotation and mirror flipping, to increase the number of training samples. After these processes, we obtained a total of 1512 samples, with 77% of them being positive samples of landslides. Then, we randomly divided these samples into a training set with 73% of the samples and a test set with 27% of the samples.

### 2.3. Reference Models and Accuracy Evaluation

All experiments in this study were performed on GeForce GTX 1660 TI 8G GPU paired with Intel i7-10700 K CPU. We employed Pytorch as the deep learning framework. For all comparison models, we set the batch size to 10 and the initial learning rate of the network to 0.0005. Adam was used as an optimizer to train 40 epochs for the model. The cross-entropy loss function was selected as the loss function. The formula is as follows:

(2) $$Loss=-\sum_{x}\left(q\left(x\right)log\left(p\left(x\right)\right)\right)$$

where *x* is the number of categories (*x* = 2 in this study), $p\left(x\right)$ is the predicted value, and $q\left(x\right)$ is the true value.

To evaluate the performance of different models in landslide deformation detection, we calculated the precision, recall, F1-score, mean intersection over union (*mIOU*), and Kappa to provide a more comprehensive evaluation of the models. These metrics are defined as follows:

Precision represents the proportion of true positive samples among the results predicted as positive by the model, reflecting the accuracy and quality of the model’s predictions when it comes to positive instances.

(3) $$Precision=\frac{TP}{TP+FP}$$

Recall measures the model’s ability to identify positive instances. When the proportion of false negatives is high, recall becomes an important evaluation indicator.

(4) $$Recall=\frac{TP}{TP+FN}$$

F1-score combines precision and recall, suitable for scenarios requiring a balance between them, providing a holistic evaluation of model performance, particularly valuable for imbalanced datasets.

(5) $$F1-score=\frac{2\times Precision\times Recall}{Precision+Recall}$$

Mean intersection over union (*mIOU*) is a commonly used metric for semantic segmentation models. It assesses overall segmentation accuracy by considering pixel-level overlap across different categories and quantifying the segmentation quality for each category.

(6) $$mIOU=\frac{1}{2}\times (\frac{TP}{TP+FN+FP}+\frac{TN}{FP+TN+FN})$$

Kappa measures the consistency between predicted results and actual labels in classification or segmentation tasks. In cases of unbalanced category distribution or uncertain labels, it provides a more robust evaluation than accuracy.

(7) $$Kappa=\frac{Par-Pre}{1-Pre}$$

(8) $$Par=\frac{TP+TN}{TP+FP+FN+TN}$$

(9) $$Pre=\frac{\left(TP+FP)\times (FN+TP)+(FN+TN)\times (FP+TN\right)}{TP+FP+FN+TN}$$

## 3. Experiment and Data processing

### 3.1. Study Area and Datasets

**Figure 4:**
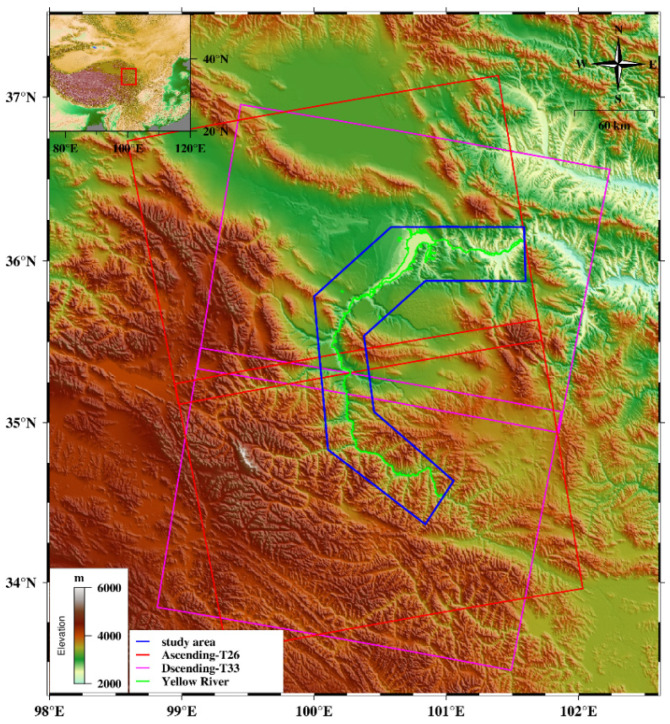
Geographical location and image coverage of the study area. The red and pink rectangles represent the coverage of Sentinel-1 ascending and descending track data, respectively. The blue polygon represents the scope of the study area, and the green line represents the Yellow River. The background image is from SRTM DEM.

The study area is located in the middle and upper reaches of the Yellow River in eastern Qinghai Province, China (34.37°–36.25° N, 100.00°–101.60° E). The altitude ranges between 2100 and 4700 m, with an average altitude of approximately 3200 m. The regional surface evolution is shaped by the north-eastward extrusion of the Qinghai–Tibet Plateau, resulting in the formation of mountainous and basin terrains [38]. The prolonged erosion and incision processes by the Yellow River and its tributaries have created favorable geomorphic conditions for landslide formation. The specific location of the study area and Sentinel-1 data coverage are shown in Figure 4. This region is characterized by active fault zones, large height differences, and rugged terrain [39]. The study area experiences a typical arid–semi-arid continental plateau climate, with the annual average precipitation of 316–436 mm, mainly concentrated in summer and autumn. The unique climatic conditions contribute to extensive rock weathering and sparse vegetation coverage [40]. These factors lead to frequent landslides in the study area, with a wide distribution. It is an ideal experimental place to validate the proposed intelligent wide-area landslide detection algorithm.

**Figure 5:**
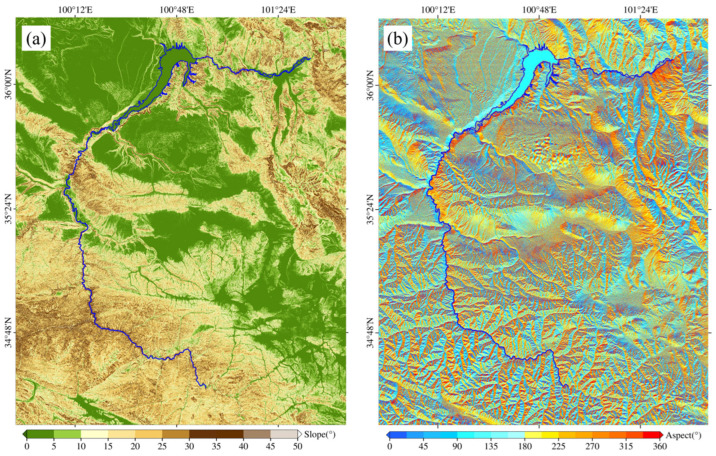
(**a**) Slope map and (**b**) slope aspect map of the study area.

We used the Sentinel-1 C-band SAR images from Ascending Track 26 (AT26) and Descending Track 33 (DT33) collected from January 2018 to April 2020. There are 57 images from AT26 and 69 images from DT33 (Table 1). The data parameter details are shown in Table 1. In addition, we used a 30 m resolution digital elevation model (DEM) from the Shuttle Radar Topography Mission (SRTM). We also calculated the slope and slope aspect (Figure 5) of the study area through DEM.

### 3.2. Data Processing

**Figure 6:**
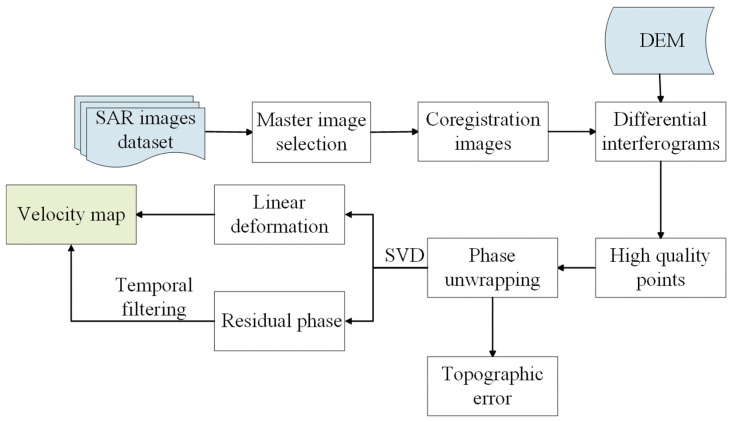
Flowchart of the MT-InSAR data processing.

**Figure 7:**
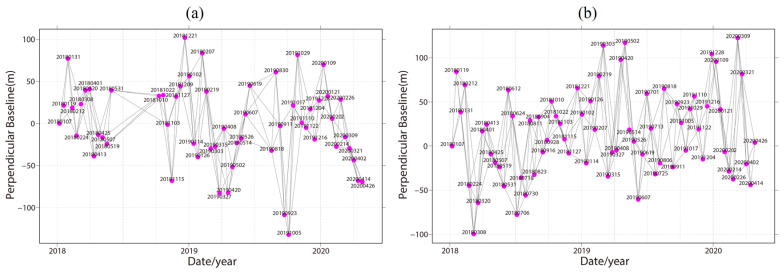
Spatiotemporal baseline network of (**a**) Track 26 and (**b**) Track 33.

We used SBAS-InSAR [41] (Figure 6) to obtain surface deformation. First, we selected SAR images covering the study area and registered them with the master image. Given the relatively small perpendicular baselines of Sentinel-1 images, we selected interferometric pairs based on the temporal baseline threshold (Figure 7). In this process, the maximum temporal baseline was set as 36 days. We used GAMMA(v 4.9) software [42] to conduct the interferometric process. A multi-look operation of 5: 1 (range: azimuth) was applied to generate interferograms. The Shuttle Radar Topography Mission (SRTM) DEM with a resolution of 30 m was employed to remove the topographic phase. Then, we set the coherence threshold as 0.3 to remove those poor-quality pixels. Subsequently, the interferograms were processed through filtering, phase unwrapping, and phase ramp removal. After that, the differential interferometric phase $\varphi_{dint}$ of each pixel can be expressed by Formula (10):

(10) $$\varphi_{dint}=\frac{4\pi B^{⊥}}{\lambda Rsin\theta }\Delta Z+\frac{4\pi }{\lambda }\Delta T\cdot v+\varphi_{res}$$

where $B^{⊥}$ is the perpendicular baseline; R is the slant range; θ is the incident angle; ΔZ is the terrain residual; ΔT is the time difference between the two images; v is the linear deformation rate; $\varphi_{res}$ is the residual phase, in which the main components are atmospheric delay, nonlinear deformation, and noise phase.

Formula (11) is the matrix representation of Formula (10). To calculate the deformation time series and topographic error of each pixel, we used the singular value decomposition (SVD) method to solve Formula (11).

(11) $$\frac{4\pi }{\lambda }\left[\begin{array}{c} \begin{array}{ccc} t_{2}-t_{1} & t_{3}-t_{2} & 0\\ 0 & t_{3}-t_{2} & t_{4}-t_{3} \end{array}\;\;\;\;\;\begin{array}{ccc} \cdots & 0 & 0\\ \cdots & 0 & 0 \end{array}\;\;\;\begin{array}{c} \frac{B^{⊥1}}{R_{1}sin\theta }\\ \frac{B^{⊥2}}{R_{2}sin\theta } \end{array}\;\;\\ \begin{array}{ccc} \vdots & \vdots & \vdots \\ 0 & 0 & 0 \end{array}\;\;\;\;\;\begin{array}{ccc} \vdots & \vdots & \vdots \\ \cdots & t_{n}-t_{n-1} & t_{n+1}-t_{n} \end{array}\;\;\;\;\;\begin{array}{c} \vdots \\ \frac{B^{⊥n}}{R_{n}sin\theta } \end{array} \end{array}\right]\left[\begin{array}{c} v_{1}\\ v_{2}\\ \vdots \\ v_{n}\\ ∆z \end{array}\right]=\left[\begin{array}{c} \varphi_{dint1}\\ \varphi_{dint2}\\ \vdots \\ \varphi_{dintn} \end{array}\right]$$

The deformation rate and topographic error of each pixel in each time period can be calculated by Formula (11). The initial deformation time series can be obtained by the integration of time differences and deformation rates. The Least Squares (LS) method was used to estimate the linear deformation rate of each pixel. The residual phase can be obtained by subtracting the linear deformation and topographic error from the initial deformation time series. The nonlinear deformation can be extracted from the residual phase by spatial and temporal filtering. The final deformation time series was derived by combining the nonlinear deformation with the linear deformation. Finally, the average deformation rate of each pixel can be calculated by the LS method.

## 4. Result and Analysis

### 4.1. Ablation Experiment

To assess the impact of integrating feature extraction and fusion modules in improving the model’s ability to identify landslides, we conducted ablation experiments using the three established datasets: Image + DEM, Image + Aspect, and Image + Slope. Table 2 shows the results. In this table, Only image indicates the absence of feature extraction and fusion modules, and only the wrapped deformation result is used. Feature fusion represents the presence of only the feature fusion module. Feature extraction + Feature fusion represents the addition of both the pseudo-Siamese network for feature extraction and the feature fusion module.

The results show that using Only image for detection yielded the lowest performance score across all evaluation indicators. When we added the feature fusion module and utilized fused data for detection, the evaluation results improved, demonstrating the effectiveness of integrating topographic data in enhancing the model’s landslide identification ability. Furthermore, with the addition of both the Feature extraction and Feature fusion modules, the performance was further enhanced. In all the experiments, the model achieved the best identification performance when using the Image + Slope dataset. Among them, the results of the four evaluation indicators recall, F1-score, *mIOU*, and Kappa were the highest in this experimental group. Although its precision did not reach the highest value, it was only 0.23% lower than the highest precision value. Compared to the results of the Only image, precision increased by 2.09%, recall by 4.76%, F1-score by 3.44%, *mIOU* by 2.98%, and Kappa by 3.54%. The ablation experiments validate that the improved network proposed in this study can extract useful features from topographic data and then fuse them with texture features from the wrapped deformation results at a higher level, thus significantly improving detection performance.

**Figure 8:**
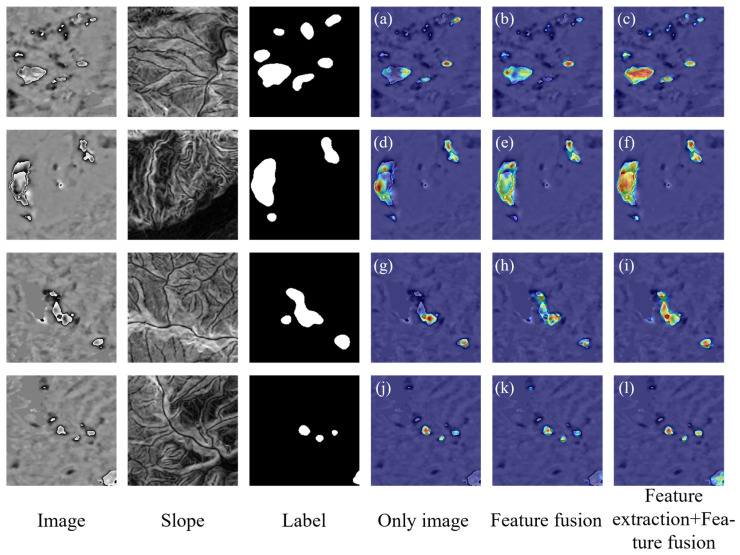
Grad-CAM visualization results of landslides in the Image + Slope dataset. The visualization results of identifying four different regions using three methods are shown in (**a**–**l**).

Next, we visualize three methods—Only image, Feature fusion, and Feature extraction + Feature fusion—using the combined data of Image + Slope. We employ the Gradient-weighted Class Activation Mapping (Grad-CAM) to visualize the regions of interest for the models [43]. We show four sets of visualization results in Figure 8. In the fourth column, the Only image method exhibits significant shortcomings in identifying the boundaries of large landslides (Figure 8a,d,g), with some boundaries not being fully recognized. There are also omissions in detecting landslides located at the edge of the sample (Figure 8j) and landslides with small deformation ranges (Figure 8a,g). The fifth column shows the results of using the Feature fusion method, which obtains relatively complete landslide boundaries. However, for landslides located at the edge of the sample (Figure 8k), this method still fails to accurately capture the deformation. In the results of the sixth column using the Feature extraction + Feature fusion method, more accurate and complete landslide boundaries are obtained. This further validates that the improved network can extract valuable information from topographic data, enhancing the accuracy of landslide identification.

### 4.2. Comparison of the Proposed Method with Other Networks

To assess the effectiveness of the proposed MSFD-Net model, we compared it with U-Net [44], FCN [45], and SegNet [46], which are widely used in semantic segmentation tasks. This comparison allows us to assess the performance of different network architectures applied to the same task. To conduct a controlled experiment, we added the feature extraction and feature fusion modules to these three networks in the same way. We trained all networks under the same experimental settings. Based on the experimental results in Section 4.1, we selected the Image + Slope dataset for comparative experiments. The results are presented in Table 3.

We set up two sets of experiments. The first group was a single-channel network using Only Image data, while the second group was a dual-channel network that incorporating the pseudo-Siamese network and utilized the Image + Slope data. The results of the second group of experiments were much better than those of the first group. Notably, in the two control experiments, the evaluation metrics associated with the MSFD-Net model in the second group achieved high scores: accuracy of 92.60%, recall of 92.31%, F1-score of 92.45%, *mIOU* of 92.69%, and Kappa value of 92.16%. The evaluation metrics of U-Net, SegNet, and FCN were all lower than those of the proposed model in this study. Among them, SegNet exhibited the lowest scores, almost all below 80%. The performance of U-Net and FCN was better than SegNet, which may be due to their use of skip connections that combine low-level and high-level features, enabling the decoding path to have more spatial information, thus improving segmentation performance.

We also compared the complexity of the models by setting the input tensor size to (1, 1, 224, 224). Table 4 shows the Params, GFLOPs, and Size of each model. MSFD-Net is a more lightweight model compared to other models, with all parameters being the smallest among the models, achieving only 4.08 M parameters and 90.71 G GFLOPs. This indicates that it has the fewest training parameters and the highest computational efficiency during the training process, yet it achieves the highest accuracy. In addition, this experiment further demonstrates that adding feature extraction and fusion network and using topographic data can improve the model’s ability to identify landslides.

### 4.3. Application of the Proposed Method to the Study Area

**Figure 9:**
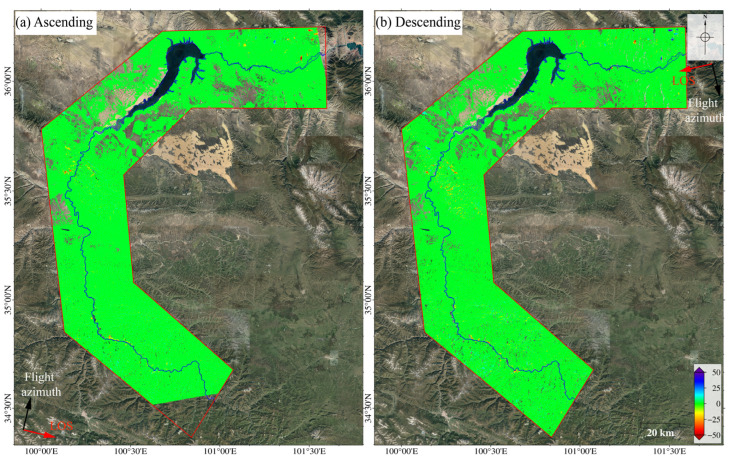
Surface deformation results based on (**a**) the ascending data and (**b**) the descending track data of the middle and upper reaches of the Yellow River from March 2018 to April 2020 obtained by SBAS-InSAR. The black and red arrows indicate the satellite’s flight direction and line of sight (LOS), respectively.

We used the method described in Section 3.2 to process the Sentinel-1 images. The obtained deformation results of the study area are shown in Figure 9. We obtained 6,140,453 coherent target points in the ascending surface deformation results and 6,576,338 in the descending surface deformation results, averaging 544 and 583 coherent target points per square kilometer, respectively. In Figure 9, most of the areas are stable, except for some notable deformation areas along the Yellow River. Among them, the maximum annual average deformation rate in the ascending result reaches 93 mm/yr and, in the descending result, reaches 78 mm/yr.

We convert the obtained deformation rate into wrapped deformation by rewrapping. Following the study of Zhao et al. [30], we selected wrap intervals of 3 cm/yr and 4 cm/yr for the ascending and descending deformation results, respectively, based on the deformation rate results. We output the wrapped deformation result as a grayscale image and normalized the pixel values to [0, 255]. Given the superior detection performance achieved with the Image + Slope dataset in our experiments, we employed this data combination for landslide detection in the study area. To prevent information loss, we segmented the data into 224 × 224 patches with a 40% overlap and input them into the pre-trained model for detection (Figure 1a). The identification results were spliced into a binary image of the same size as the wrapped deformation result. The reasonable boundary of suspected landslides was determined by dilation, smoothing, and edge detection (Figure 1c). Due to the unique side-looking geometry of SAR satellites, different observation angles yield different deformation rate results [26]. Therefore, we chose to merge the identification results from ascending and descending tracks to improve the detection rate of landslides.

**Figure 10:**
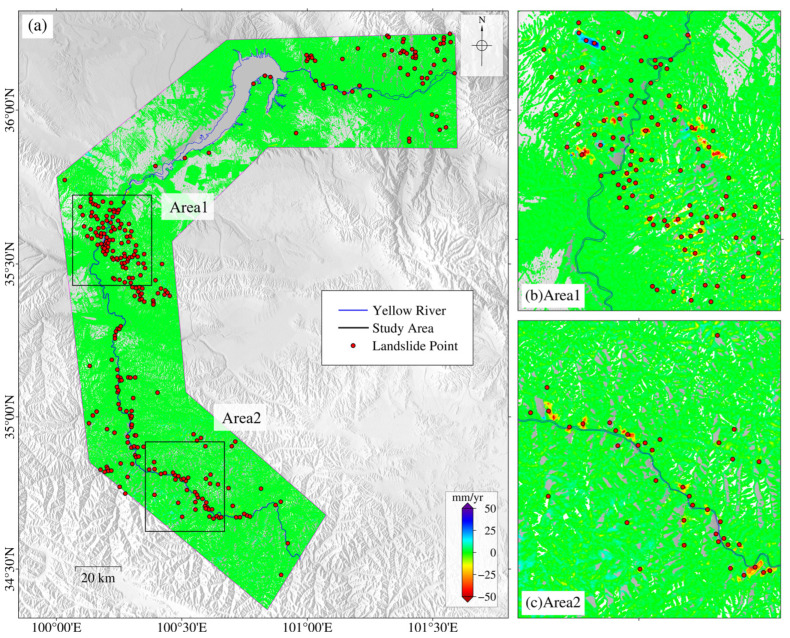
**The suspected landslide points identified by the proposed method shown in (a).** Red dots denote the suspected landslide points, and the blue line is the Yellow River. Zoomed-in images of two areas identified by the black rectangles are shown in (**b**,**c**).

**Figure 11:**
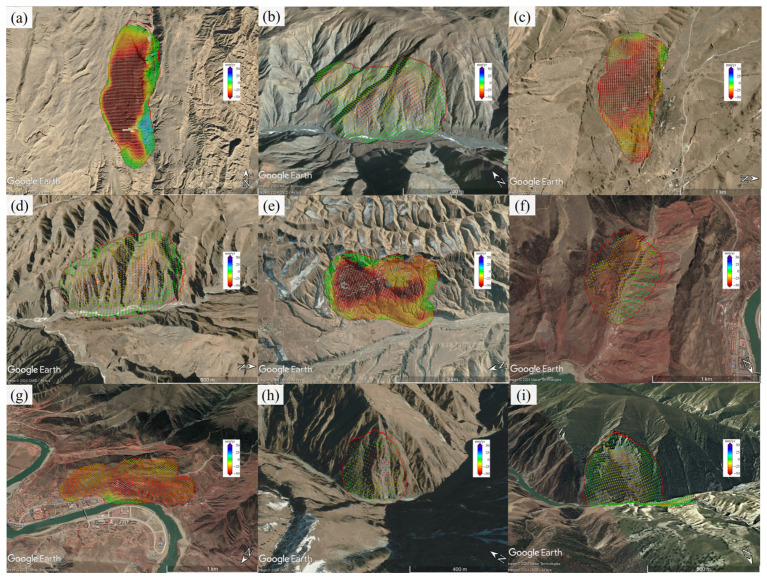
Google Earth™ images of some landslides detected in the study area are shown in (**a**–**i**). The red polygons delineate the identified landslide boundary.

Finally, we identified a total of 298 suspected landslide points in the study area, with the distribution map of landslide points shown in Figure 10. Among them, 165 were identified in the ascending track deformation, and 200 in the descending track deformation. To further verify these suspected landslide points, we superimposed the identification results on Google Earth^TM^. The optical images and deformation results of some landslides are shown in Figure 11. Using our experience in identifying landslides from optical images, we confirmed 254 active landslides based on their morphological features in the optical images. The average area of these landslide points was 0.44 km^2^, with a minimum area of 0.04 km^2^ and a maximum area of 4.24 km^2^. The remaining points could not be classified as landslides, resulting in an accuracy of approximately 85% in this study area. Overall, landslides are mostly concentrated on both sides of the Yellow River basin, particularly in areas with a certain slope gradient. Figure 10b,c show some of the identified landslide points.

## 5. Discussion

### 5.1. Distribution of Landslide Points in the Study Area

**Figure 12:**
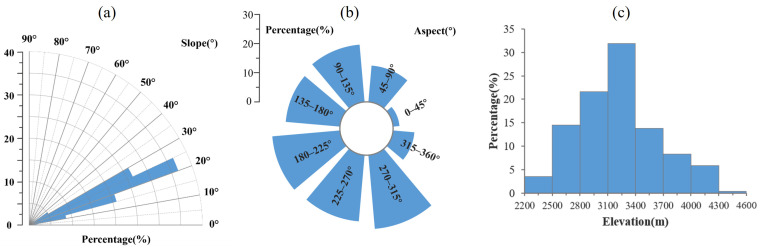
Statistical percentage of landslides by (**a**) slope gradient, (**b**) slope aspect, (**c**) elevation.

The occurrence of landslides is associated with natural factors such as geological structure, hydrological conditions, terrain features, and earthquakes. Notably, topographic features play a crucial role in landslide formation, including slope gradient, slope aspect, and elevation [47]. Therefore, we conducted a quantitative statistical analysis of the relevant topographic features of the 254 landslide points identified in Section 4.3. The specific results are shown in Figure 12.

We divided the slope gradient into eight intervals to analyze the distribution of identified landslide slope gradients, following the research of [48]. As shown in Figure 12a, among the 254 landslide points in the study area, 92.52% were associated with slope gradient between 10° and 30°. This indicates that when the slope gradient of an unstable slope satisfies certain conditions, it can overcome the friction strength of the sliding surface, leading to a landslide, when combined with external environmental influences. The occurrences of landslides in areas with a slope gradient <10° and 30–40° are relatively infrequent. Notably, no landslides were observed in areas with slope gradient >40° in this study area. Slopes with gradients less than 10° exhibit gentle topography and small potential energy, insufficient to overcome the frictional strength of the sliding surface. Over time, these slopes are gradually eroded by rainfall and weathering, forming a gentle slope that adapts to its strength [49]. Therefore, when the slope gradient is less than 10°, the slope is not prone to landslide movement, resulting in a low probability of occurrence. When the slope gradient is >40°, the probability of landslides decreases sharply. This can be explained by the fact that slope gradient is not the only factor affecting landslide occurrence, and the higher slope gradients do not necessarily correlate with increased landslide probabilities. A steeper slope brings higher internal rock strength, providing higher control over the interior. Additionally, when the slope is steep, the kinetic energy of the slope exceeds its friction strength; steeper slopes may experience material shedding processes like rockfall and collapse to maintain energy balance [48].

We divided slope aspect into eight intervals, each spanning 45°. As shown in Figure 12b, the average slope aspect of the identified landslide is mainly concentrated between 90° and 315°, with a uniform distribution, while relatively fewer landslides are distributed in the 0–45°, 45–90°, and 315–360°. We further divided the elevation range of 2200–4600 m into eight intervals of 300 m each. As shown in Figure 12c, approximately 81.89% of landslides are distributed at an altitude of 2500–3700 m. The incidence of landslides increases between 2200 m and 3400 m but decreases gradually above 3400 m. Additionally, the density of landslides in the study area is the highest between 2800 and 3400 m. Our findings align with the landslide detection results from the upper reaches of the Yellow River in 2023 [40].

### 5.2. The Impact of Topographic Data on Landslide Identification

The occurrence of landslides is closely related to topographic factors, and the integration of such data can effectively enhance the accuracy of landslide identification [50]. In this study, we trained and validated the model using slope, slope aspect, and DEM data. As shown in Table 2, the identification accuracy of integrating topographic data was better than that without it. Moreover, among various evaluation indicators, the addition of slope data yielded the best performance. Generally, slope gradient has a high correlation with landslide occurrence. Steeper slopes inherently possess greater potential energy, making them more susceptible to landslides due to increased sliding forces [51,52].

Slope aspect significantly influences landslide occurrence, primarily through natural factors, such as sunlight, precipitation, and erosion. These factors impact soil moisture, vegetation coverage, and the stability of the slope, thereby affecting the occurrence of landslides [53]. As shown in Section 5.1, the slope aspect distribution in the study area is relatively uniform. Therefore, the correlation between the slope aspect and landslides is less significant compared to slope gradient. Landslides generally occur in areas with high altitudes and large elevation differences. High-altitude areas often have steep terrain, exposed rocks, and weak soil, all of which may increase the probability of landslides [54]. In addition, elevation changes may also affect geological structures such as faults and rock dip angles, further influencing the formation and distribution of landslides. However, in the area with a small elevation difference, the influence of elevation on landslides is not obvious. Although using slope aspect and elevation data can improve the accuracy of landslide identification, the effect is not as significant as when using the slope gradient data.

**Figure 13:**
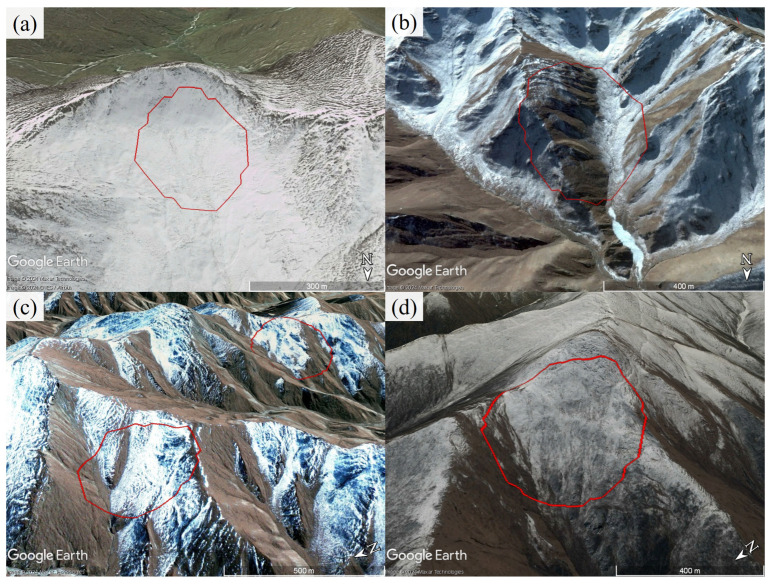
Optical images of false positive results caused by melting glaciers and snow are shown in (**a**–**d**). The red polygon is the deformation range identified by the proposed method.

The mechanism behind landslide formation is complex, and relying solely on single topographic data cannot achieve perfect accuracy in landslide identification. We found that 31 deformation areas (identification results in Section 4.3) were caused by the melting of glaciers and snow in high-altitude regions (Figure 13). There are also deformation areas caused by human activities that are misinterpreted as landslides, such as engineering construction zones on slopes and agricultural irrigation areas. These areas exhibit deformation characteristics akin to landslide deformation, leading to false positives. Such misclassification is acceptable, as we aim to identify potential landslides as comprehensively as possible. In addition, data resolution also has a significant impact on landslide identification. High-resolution data can capture subtle deformation texture features and more topographic features, providing more detailed information to improve the landslide identification accuracy. However, excessively high resolution may lead to noise or excessive detail. And low resolution may not provide rich information for identification. Therefore, selecting an appropriate data resolution is crucial for improving the effectiveness of landslide identification. Additionally, the accuracy of landslide identification is also affected by the limitations of sample size and diversity. Some samples are labeled based on expert experience, introducing subjectivity and potentially deviating the identification results to an accurate extent. Although this study has improved the accuracy of landslide identification by fusing topographic features, the complex nature of landslide occurrence mechanisms suggests avenues for further improvement. In the future, we can try to increase the number of fusion channels or introduce more data affecting landslide occurrence, such as geological and rainfall data, to further enhance the precision of landslide identification.

## 6. Conclusions

In this study, we proposed a multi-source data fusion network based on DeforNet for landslide detection, which combines the topographic conditions of landslide occurrence. This network improves the existing DeforNet geological hazard detection network by adding a pseudo-Siamese network without weight sharing. It can effectively extract texture features from the wrapped deformation results and topographic features from topographic data, which are then fused at higher feature levels. We demonstrated through ablation experiments that the performance of the improved network was enhanced. We used Sentinel-1 SAR data covering the upper and middle reaches of the Yellow River from January 2018 to April 2020. The deformation rate results were obtained by the SBAS-InSAR technology. Then, using the proposed method, we identified 165 suspected landslides in the ascending track and 211 in the descending track, with a minimum landslide area of 0.04 km^2^. Through quantitative analysis of the topographic features associated with landslides in the study area, we observed a correlation between landslide occurrence and terrain, with slope being a key factor affecting landslide generation. The slope range of 10–30° is the main slope range for landslide development, accounting for 92.28%.

InSAR provides a large amount of data for the identification of wide-area landslides, and the deep learning method improves the identification efficiency. The introduction of topographic data through multi-source data fusion can provide more valuable information, thus improving the efficiency and accuracy of landslide identification in complex surface deformations. The intelligent detection method proposed in this study provides greater convenience for low-cost, large-area, efficient, and accurate wide-area InSAR landslide identification.

## Figures and Tables

**Table 1:** Data information.

Sensor	Band (Wavelength/cm)	Track Direction	Orbit	Pixel Spacing (Rg × Az)	Incidence Angle	Heading Angle	Number of Scenes
Sentinel-1	C (5.6)	Ascending	T26	2.33 × 13.96 m	40.76°	13.08°	57
	C (5.6)	Descending	T33	2.33 × 13.96 m	39.43°	193.08°	69

**Table 2:** Comparative results of feature fusion scheme.

Method and Data		Precision (%)	Recall (%)	F1 (%)	mIOU (%)	Kappa (%)
Only image		90.51	87.55	89.01	89.71	88.62
Feature fusion	Image + DEM	91.89	90.46	91.17	91.55	90.83
	Image + Aspect	91.53	89.31	90.41	90.88	90.04
	Image + Slope	**92.83**	89.16	90.96	91.36	90.61
Feature extraction + Feature fusion	Image + DEM	92.24	90.17	91.19	91.57	90.85
	Image + Aspect	91.80	91.85	91.82	92.12	91.50
	Image + Slope	92.60	**92.31**	**92.45**	**92.69**	**92.16**

Note: Numbers in bold are the best results for each indicator.

**Table 3:** Performance of the four networks in identifying landslides.

Data	Method	Precision (%)	Recall (%)	F1 (%)	mIOU (%)	Kappa (%)
Only image	U-Net	89.98	84.98	87.41	88.34	86.93
	SegNet	82.45	64.57	72.42	77.43	71.48
	FCN	89.12	87.82	88.47	89.21	88.02
	MSFD-Net	90.51	87.55	89.01	89.71	88.62
Image + Slope	U-Net	90.70	87.97	89.32	89.94	88.91
	SegNet	76.15	76.52	76.33	79.94	75.41
	FCN	90.55	86.85	88.66	89.38	88.23
	MSFD-Net	**92.60**	**92.31**	**92.45**	**92.69**	**92.16**

Note: Numbers in bold are the best results for each indicator. Image represents the wrapped deformation result.

**Table 4:** Trainable parameters and complexity of models.

Method	Params (M)	GFLOPs (G)	Size (MB)
U-Net	17.31	32.37	66.04
SegNet	29.48	32.36	112.49
FCN	20.14	18.73	76.85
MSFD-Net	4.08	9.06	15.55

## Data Availability

The copyright of Sentinel-1 data used in this study is owned by the European Space Agency (https://search.asf.alaska.edu/, accessed on 1 September 2023). The GAMMA commercial software was obtained from https://www.gamma-rs.ch/software, accessed on 22 August 2023.
